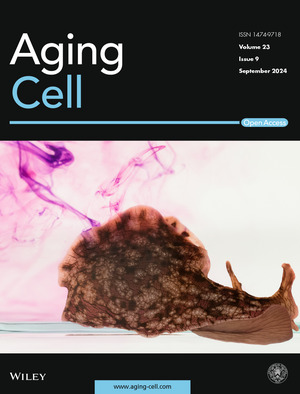# Additional Cover

**DOI:** 10.1111/acel.14347

**Published:** 2024-09-12

**Authors:** Kerriann K. Badal, Abhishek Sadhu, Bindu L. Raveendra, Carrie McCracken, Sebastian Lozano‐Villada, Amol C. Shetty, Phillip Gillette, Yibo Zhao, Dustin Stommes, Lynne A. Fieber, Michael C. Schmale, Anup Mahurkar, Robert D. Hawkins, Sathyanarayanan V. Puthanveettil

## Abstract

Cover legend: The cover image is based on the Article *Single‐neuron analysis of aging‐associated changes in learning reveals impairments in transcriptional plasticity* by Kerriann K. Badal et al., https://doi.org/10.1111/acel.14228 Image Credit: Phillip Gillette and Lynne Fieber